# Three-Dimensional Graphene–RGD Peptide Nanoisland Composites That Enhance the Osteogenesis of Human Adipose-Derived Mesenchymal Stem Cells

**DOI:** 10.3390/ijms19030669

**Published:** 2018-02-27

**Authors:** Ee-Seul Kang, Da-Seul Kim, Yoojoong Han, Hyungbin Son, Yong-Ho Chung, Junhong Min, Tae-Hyung Kim

**Affiliations:** 1 School of Integrative Engineering, Chung-Ang University, Seoul 06974, Korea; eeseul94@cau.ac.kr (E.-S.K.); dptmf4011@cau.ac.kr (D.-S.K.); handbwnd@naver.com (Y.H.); being@cau.ac.kr (H.S.); 2 Department of Chemical Engineering, Hoseo University, Asan City, Chungnam 31499, Korea; yhchung@hoseo.edu; 3 Integrative Research Center for Two-Dimensional Functional Materials, Institute of Interdisciplinary Convergence Research, Chung-Ang University, Seoul 06974, Korea

**Keywords:** graphene oxide, silica nanoparticles, gold nanoparticles, RGD peptide, differentiation, mesenchymal stem cells, adipose-derived stem cells, osteogenesis

## Abstract

Graphene derivatives have immense potential in stem cell research. Here, we report a three-dimensional graphene/arginine-glycine-aspartic acid (RGD) peptide nanoisland composite effective in guiding the osteogenesis of human adipose-derived mesenchymal stem cells (ADSCs). Amine-modified silica nanoparticles (SiNPs) were uniformly coated onto an indium tin oxide electrode (ITO), followed by graphene oxide (GO) encapsulation and electrochemical deposition of gold nanoparticles. A RGD–MAP–C peptide, with a triple-branched repeating RGD sequence and a terminal cysteine, was self-assembled onto the gold nanoparticles, generating the final three-dimensional graphene–RGD peptide nanoisland composite. We generated substrates with various gold nanoparticle–RGD peptide cluster densities, and found that the platform with the maximal number of clusters was most suitable for ADSC adhesion and spreading. Remarkably, the same platform was also highly efficient at guiding ADSC osteogenesis compared with other substrates, based on gene expression (alkaline phosphatase (ALP), runt-related transcription factor 2), enzyme activity (ALP), and calcium deposition. ADSCs induced to differentiate into osteoblasts showed higher calcium accumulations after 14–21 days than when grown on typical GO-SiNP complexes, suggesting that the platform can accelerate ADSC osteoblastic differentiation. The results demonstrate that a three-dimensional graphene–RGD peptide nanoisland composite can efficiently derive osteoblasts from mesenchymal stem cells.

## 1. Introduction

Stem cells have emerged as a promising source in the field of regenerative medicine, because of their innate ability to produce a variety of desired cell types in the human body [1,2]. Among stem cells, mesenchymal stem cells (MSCs) have predominated in clinical trials and commercialization, since they are relatively easy to obtain from various sources (e.g., adipose tissue, bone marrow, dental tissue), free from ethical issues, and most importantly, have a low risk of teratoma formation after transplantation [3]. MSCs are also attractive because of their capability to generate various types of cells (e.g., osteoblasts, chondrocytes, adipocytes), all of which are fundamental for the reconstruction and regeneration of most body components [4,5,6]. However, to maximize regeneration efficiency, the characteristics of MSCs should be properly adjusted to the desired cell type through differentiation, because their multipotency may also result in the production of unwanted cell types [7,8,9].

The differentiation of stem cells is conventionally controlled by soluble cues; that is, the use of defined culture media containing multiple growth factors (e.g., bone morphogenetic proteins, insulin, peroxisome proliferator-activated receptor γ) and biomolecules (e.g., dexamethasone, ascorbic acid, glycerophosphate) [10,11,12,13,14]. However, recent findings suggest that biophysical cues, such as the physicochemical characteristics of the underlying substrate, to which the cells attach and actively interact with as they grow, can also play key roles in steering the transformation of MSCs into specific cell types [15,16,17,18]. Mechanical stimulation, a biophysical cue that can affect cell behavior, is generally initiated by cell–substrate interactions, which alter cytoskeletal dynamics and consequently affect gene expression in the cells [12,15,19,20,21,22,23,24,25]. To provide favorable biophysical stimulation to stem cells, nanostructures, micropatterns, and functional composite materials have been utilized to control stem cell behavior, particularly differentiation, with and without the use of soluble factors [26,27,28,29,30,31]. Among such materials, graphene and graphene oxide (GO) have recently been shown to effectively provide the necessary physical stimulation for stem cell differentiation [32,33,34,35,36]. The distinct physicochemical characteristics of graphene derivatives were found to successfully initiate several specific MSC differentiation routes, such as adipogenesis [37], osteogenesis [38,39], and neurogenesis [40]. The multifarious surface chemistry, controllable amphiphilicity, and unique honeycomb carbon structure were reported to affect several cellular aspects such as spreading, growth, and morphology, as well as the absorption kinetics of proteins and chemicals in the differentiation medium [32,33,41,42,43,44]. 

In addition to graphene derivatives, natural polymers such as extracellular matrix (ECM) proteins (e.g., fibronectin, laminin, and vitronectin) and peptides are also excellent candidates for controlling stem cell behavior [45,46]. Among the various available materials, peptides containing a repeating arginine–glycine–aspartic acid (RGD) sequence, which is often found in ECM proteins, have been widely applied not only to increase the uptake of materials and molecules of interest into specific types of cells, but also to enhance cell adhesion to artificial platforms such as synthetic polymers and metallic surfaces [47,48,49]. Interestingly, RGD-containing peptides immobilized onto substrates enhanced the adhesion strength of MSCs, ultimately resulting in increased osteogenic differentiation, as cell adhesion is a key factor guiding osteogenic lineage commitment [50,51]. 

**Figure 1:**
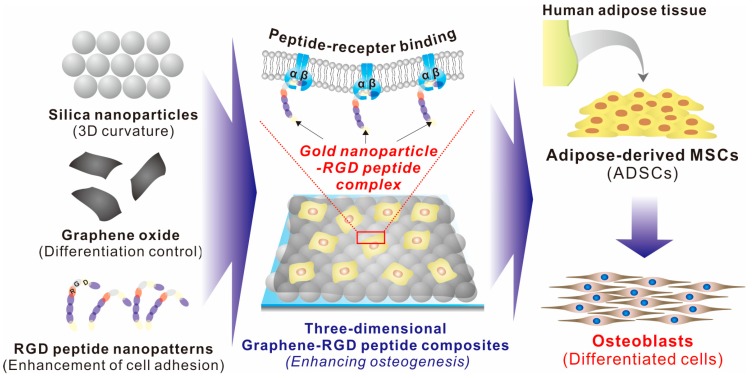
Schematic diagram of three-dimensional graphene/arginine-glycine-aspartic acid (RGD) peptide composites for enhancement of adipose-derived mesenchymal stem cell (ADSC) osteogenesis.

In this study, we report a three-dimensional graphene/RGD peptide nanoisland composite that is highly effective in controlling the osteogenic differentiation of human-adipose-derived mesenchymal stem cells (ADSCs; Figure 1). ADSC differentiation was controlled by four independent factors: (i) GOs that contribute to the absorption of several differentiation factors; (ii) RGD–MAP–C peptides that enhance cell adhesion; (iii) nanopatterns with randomly-distributed geometry and iv) height variations of underlying substrates that facilitate cell–GO and cell–peptide interactions (Figure 1). The physical and chemical properties of the three-dimensional nanocomposites were first characterized by X-ray photoelectron spectroscopy (XPS) and Raman spectroscopy. Thereafter, ADSCs were cultivated on GO surfaces with varying numbers of nanoparticle–RGD peptides and their adhesion, spreading, and proliferation were analyzed. Furthermore, the effects of nanoparticle–RGD peptides complexes on ADSC osteogenesis were characterized by assaying mRNA expression and calcification levels. Finally, to better understand the effects of nanoparticle–RGD peptides on the osteogenic differentiation of ADSCs, a four-week time course was performed in the presence of culture medium with and without differentiation factors. 

## 2. Results

### 2.1. Fabrication of Silica-Nanoparticle–Graphene-Oxide Composites on an Indium Tin Oxide (ITO) Substrate

**Figure 2:**
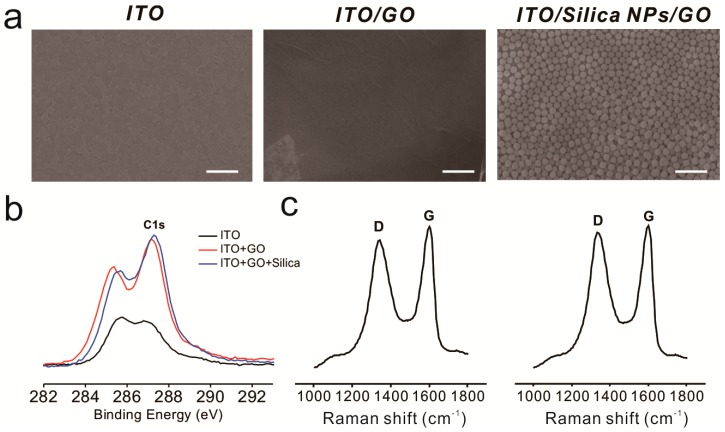
Characterization of the basic substrates. (**a**) Scanning electron microscopy images of indium tin oxide (ITO) (left), ITO/graphene oxide (GO) (middle), and ITO/GO/silica nanoparticles (SiNPs) (right). Scale bars = 1 μm; (**b**) X-ray photoelectron spectroscopy (XPS) results for each substrate; (**c**) Raman spectroscopy data for ITO/GO (left) and ITO/GO/SiNPs (right).

To enhance cell–substrate interactions, we hypothesized that the generation of RGD nanopattern ripples on the graphene surface would facilitate both α_v_β_3_ ligand–RGD peptide and GO–cell membrane interactions, which ultimately affect stem cell behavior. There are several methods that could be utilized to fabricate three-dimensional environments on a GO surface. Photolithography is a representative tool suitable for the fabrication of various nano- and micro-structures with precisely controlled geometry [52,53]. However, this technique includes laborious and time-consuming steps such as photomask fabrication, photoresist coating, cleaning, and etching/metal deposition. Moreover, residual photoresist on the substrates could result in unexpected toxic effects on stem cells. Hence, silica nanoparticles (SiNPs), a material proven to be nontoxic and safe for use with most cell lines [54,55], were used as a coating material to generate height variations on the ITO substrate prior to GO modification. We then attached amine-functionalized SiNPs to the negatively charged GO via electrostatic interactions. As shown in Figure 2a, GO-coated SiNPs were uniformly coated onto the ITO substrate, and the ITO/GO substrate showed the typical wrinkle-like structure of graphene, different from the bare ITO substrate. In addition, XPS spectra revealed that the chemical structure of GO sheets on SiNPs were similar to those on the ITO substrate, showing C–C, C–O and C=O bonding, which are all typically found on the GO surface (Figure 2b and Figure S1). Raman spectroscopy was used to investigate GO oxidation. It has been reported that the G band represents the in-plane structure (sp^2^-bonded carbon) while the D band indicates structural defects, such as oxidized carbon. Based on this, the structural defects and carbon-to-oxygen ratio were analyzed. Figure 2c and Figure S2 show clear D (1350 cm^−1^) and G (1600 cm^−1^) peaks for ITO/SiNP/GOs, demonstrating the successful absorption of GO sheets onto amine-modified SiNP surfaces. The I_D_/I_G_ values of ITO/GOs and ITO/SiNP/GOs were calculated to be 0.96 and 0.97, indicating that both GOs were highly oxidized, with C–C and C–O/C=O/O=C–O ratios of approximately 1:1. GO sheets embedded on the surface of SiNPs not only act as a conducting layer to enable electrochemical deposition of gold nanoparticles (GNPs) onto the SiNPs, but also helped attract gold chloride ions, resulting in the formation of the seed for GNP synthesis. Hence, it can be concluded that the fabricated GO-SiNP composite is a suitable platform for the generation of GNPs via electrochemical deposition, which could be further utilized to control stem cell behavior.

### 2.2. Fabrication of a 3D Graphene–RGD Peptide Nanoisland Composite for Stem Cell Cultures

**Figure 3:**
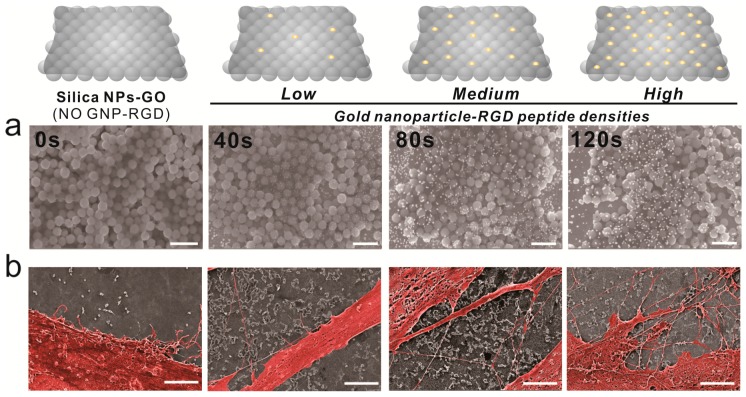
Scanning electron microscopy (SEM) images of ADSCs on gold-deposited substrates. (**a**) From left to right, SEM images of gold deposition. Scale bars = 1 μm; (**b**) Pseudocolor SEM images of ADSCs showing cell spreading. Scale bars = 3 μm.

**Figure 4:**
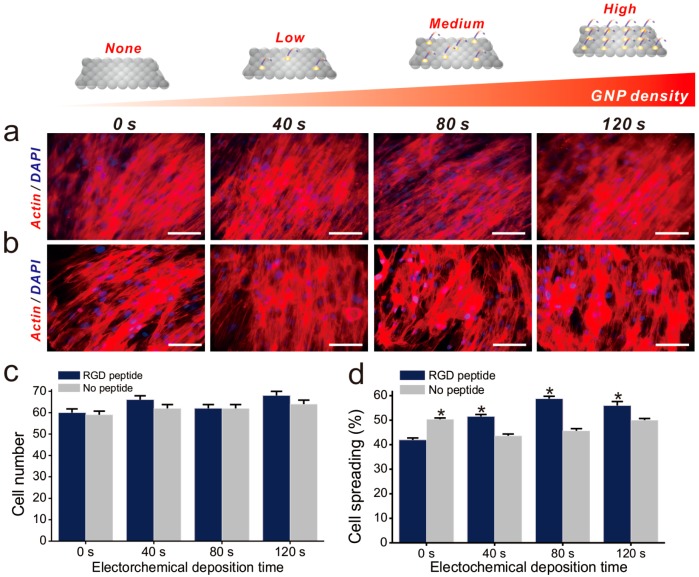
Fluorescence images of all substrates Fluorescence images of each substrate with (**a**) and without (**b**) RGD peptide. Scale bars = 100 μm; (**c**) Number of cells on each substrate after 2 day of incubation; (**d**) Spreading of proliferated cells. * Student’s *t*-test, *p* < 0.05, *n* = 3.

Cell adhesion is controlled by transmembrane glycoproteins, including integrins, selectins, and cadherins [56,57,58]. Integrins are core materials that actively regulate the anchoring of the cell membrane to the extracellular environment, and are hugely affected by the physicochemical properties of the underlying substrates [59,60]. A number of studies have reported that nanotopographic features influence many key cellular activities, including cell migration, proliferation, differentiation, and even apoptosis [61,62,63,64]. Specifically, since the size of the integrin subunits that are located in the outside of the cell membrane (α and β chains) is around 23nm and the final N-termini of each chain is around 5nm, the materials whose sizes are in the nanometer range could result in the changes of integrin-mediated cell anchoring [62,65]. Consequently, this causes cytoskeletal rearrangements of cells adhered to the nanomaterial surfaces, resulting in changes of cell signaling pathways, which ultimately affect cell growth, spreading and differentiation. In the case of MSCs, nanopattern geometry and arrangement are critical for both multipotency maintenance and differentiation. Dalby et al. reported that nanopatterns with random arrangements can effectively guide MSC osteogenesis, while the same nanopatterns with homogeneous arrangements maintain their multipotency in long-term cultures [65]. Based on this, we generated GNPs on GO-coated SiNPs via electrochemical deposition, as shown in Figure 3a. Due to the presence of the conducting GO layer, GNPs of 10–20 nm in diameter were successfully generated on the non-conducting SiNPs. Interestingly, the I_D_/I_G_ values of Raman spectra were calculated to be 1.01, 1.04, 1.18, and 1.2 after 0, 40, 80, and 120 s of deposition, respectively, indicating that GO oxidation increased with increased electrochemical deposition (Figure S2). As the RGD-containing peptides (RGD–MAP–C) contain a terminal cysteine residue, they can be easily self-assembled on the surface of the GNPs. In addition, by varying the electrochemical deposition time, the size and density of GNPs could be varied, resulting in different RGD–MAP–C peptide arrangements, which would ultimately alter the cytoskeletal dynamics of stem cells. To investigate this, ADSCs were seeded onto each platform at different GNP densities, and therefore with different RGD–MAP–C peptide arrangements. As shown in Figure 3b, the GO-SiNP-GNP-RGD peptide composites with the highest GNP density (deposition time = 120 s) showed more active filopodia interaction with the underlying platform, indicating that this substrate might effectively enhance cell adhesion and migration, improving ADSC osteogenesis. To further study the effects of GNP-RGD peptide composites on ADSCs, the substrates were divided into two groups: (i) GO-SiNP-GNPs and (ii) GO-SiNP-GNP-RGD peptide composites. After two days of culture, the cytoskeletal structures and nuclei of ADSCs were visualized by phalloidin and Hoechst 33342 staining to calculate the rates of cell spreading and growth by fluorescence microscopy (Figure 4a,b and Figure S3). Cell growth was not affected by the presence of RGD–MAP–C peptides (Figure 4c), while cell spreading was highly enhanced on all the substrates possessing GNP-RGD peptide composites (Figure 4d). Cell spreading was 118, 128, and 110% higher on GNP-RGD composites compared to GNPs only, after 40, 80, and 120 s of deposition, respectively. Cell spreading seemed to increase with increased GNP density; however, owing to the limitations in the area in which cells could attach and grow, the enhancement of cell spreading was saturated with medium GNP density. Interestingly, cell spreading was not enhanced by RGD–MAP–C peptide modifications to the GO-SiNP platform, because of the absence of GNPs, which mediate RGD–MAP–C peptide immobilization via an Au–S bond. Accordingly, based on the cell adhesion, spreading, and proliferation results, we conclude that GNP-RGD peptide modifications to the GO-SiNP platform positively affect ADSC adhesion, and can thus be considered a candidate platform to control stem cell differentiation.

### 2.3. Guiding ADSC Osteogenesis Using Graphene–RGD Peptide Nanoisland Composites

**Figure 5:**
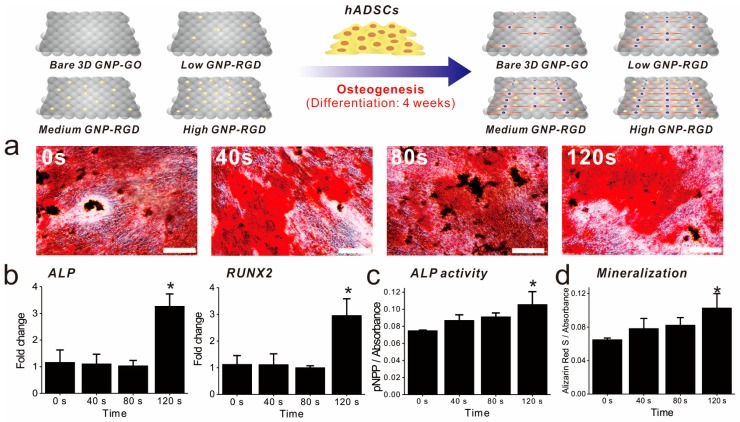
Confirmation of ADSC osteogenic differentiation. (**a**) Alizarin Red S staining of all substrates. Scale bars = 200 μm; (**b**) RT-qPCR data for alkaline phosphatase (ALP), and RUNX2; (**c**) The ALP activity of each substrate; (**d**) Absorbance rates after Alizarin Red S (ARS) staining. * Student’s *t*-test, *p* < 0.05, *n* = 3.

As cell adhesion and spreading were highly enhanced by GNP-RGD peptide modifications, we next investigated whether these differences in cell behavior affected ADSC osteogenesis. Differentiation was performed in medium containing well-known osteogenic differentiation factors (β-glycerophosphate, ascorbic acid, and dexamethasone; Figure 5). After four weeks of differentiation, ADSC osteogenesis levels were analyzed using several markers, including alkaline phosphatase (ALP) enzyme activity, ALP and runt-related transcription factor 2 (RUNX2) expression, and osteogenesis mineralization. ALP regulates the dephosphorylation of several biomolecules and is an indicator of pre-osteogenesis stem cells, while RUNX2 is critical for osteoblastic differentiation. Based on reverse transcription-quantitative polymerase chain reaction (RT-qPCR) results, remarkably, the expression of both genes was highly enhanced on GO-SiNPs with high levels of GNP-RGD peptides compared with bare 3D GO-SiNPs and the same substrate with low and medium GNP-RGD peptide densities (263% and 295% higher than low density of gold deposition for ALP and RUNX2, respectively (Figure 5b). Next, to confirm the superiority of 3D GO-RGD peptide nanoisland composites with high GNP density in ADSC osteogenic differentiation, the ALP enzyme activity and calcification levels were evaluated, using para-nitrophenylphosphate and Alizarin Red S (ARS) as colorimetric reagents, respectively. MSCs accumulation of calcium phosphate (hydroxyapatite mineral (Ca_10_(PO_4_)_6_)), an essential material for building bone structure, is an indicator of osteogenesis. As shown in Figure 5a, all ADSCs cultured in osteogenic medium successfully differentiated into cells displaying calcium deposits. Three-dimensional GO-RGD peptide nanoisland composites with high GNP density showed the best osteogenic differentiation efficiency based on ALP and ARS levels, which were 148% and 158% higher than with bare GO-SiNP platforms (Figure 5c,d). This is consistent with a previous study reporting that modifications to ECM-derived RGD-glycoproteins (e.g., fibronectin, vitronectin, and osteopontin) on cell culture substrates are critical for MSC osteogenic differentiation. Hence, it is highly likely that the increase in RGD–MAP–C peptide density in combination with the three-dimensional GO sheets on the SiNPs synergistically enhance ADSC osteogenesis via increased cell adhesion and absorption of differentiation factors. Based on these observations, we can logically conclude that the developed graphene–RGD peptide nanoislands are a promising platform to guide the differentiation of stem cells into specific lineages.

### 2.4. Time Course of ADSC Osteogenic Differentiation on Graphene–RGD Peptide Nanoislands

**Figure 6:**
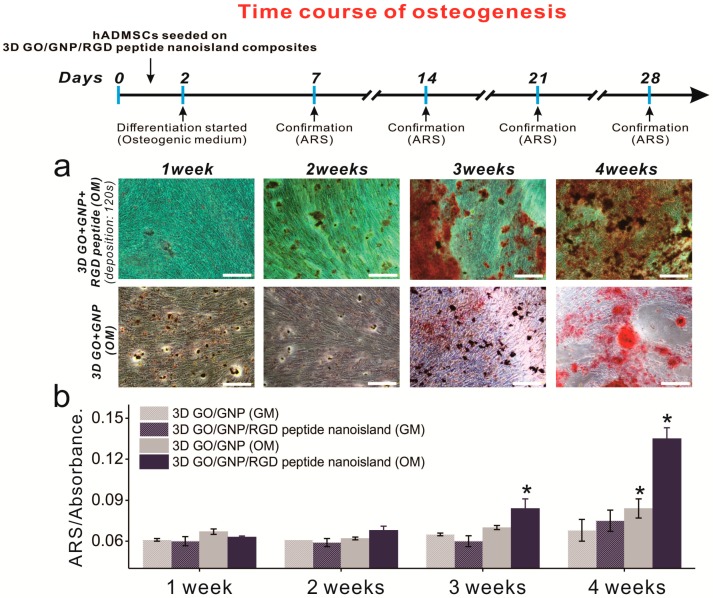
Time course of osteogenesis with and without RGD–MAP–C peptides. (**a**) ADSC differentiation was monitored every 7 d. Scale bars = 200 μm; (**b**) ARS absorbance changes in the presence of gold and different media. * Student’s *t*-test, *p* < 0.05, *n* = 3.

After confirming that the graphene–RGD peptide nanoislands with high GNP density are highly effective in guiding ADSC osteogenesis, we next investigated whether the platform could accelerate ADSC osteogenesis. This is important to study, because accelerated differentiation is needed to supply osteoblasts to the patients requiring urgent orthopedic surgery. In fact, it takes up to four weeks to generate bone cells from stem cells, and this is an obstacle in the clinical use of stem-cell-derived osteoblasts. Osteogenic ADSC differentiation was induced using typical osteogenic medium, and ARS staining was performed weekly to evaluate the osteoblastic differentiation of ADSCs grown on GO-SiNP/GNPs with and without RGD–MAP–C peptides. For the first 14 days, there was no discernable increase in osteoblast mineralization (Figure 6a). However, after two weeks of differentiation, the difference was apparent, especially between days 14 and 21 (Figure 6 and Figure S4). Specifically, the conversion of MSCs into osteoblasts on the GO-SiNP/GNP/RGD–MAP–C substrate was 120% and 160% higher than on the same substrate without GNP-RGD–MAP–C composites at days 21 and 28, respectively. Interestingly, the osteoblastic mineralization of ADSCs was highly enhanced at day 21 on the GO-SiNP/GNP/RGD–MAP–C substrate compared to on typical GO-SiNP substrates, indicating that the platform might be useful to more rapidly generate osteoblasts from stem cells, which could help meet timely clinical demands (Figure 6b). More studies regarding the gene expression levels of adhesion-related proteins (e.g., integrins, vinculin, focal adhesion kinase, paxillin, and talin) and osteogenic differentiation (e.g., osteocalcin, osteonectin, osteopontin) should be performed to validate the aforementioned results.

## 3. Discussion

In this study, we report a new platform that enables the stable long-term culture of ADSCs and guides their osteogenic differentiation. Among the various substrates fabricated, the three-dimensional GO-SiNP platform with high GNP-RGD peptide density, which we have termed the graphene–RGD peptide nanoisland composite, was best at enhancing stem cell adhesion and spreading on the artificial surface. Furthermore, this substrate was superior to other platforms, including bare GO-SiNPs and GO-SiNPs with low and medium densities of GNP-RGD peptides, at guiding ADSC osteogenesis. Specifically, the RNA expression levels of ALP (a pre-osteogenic marker) and RUNX2 (an osteogenic marker) in ADSCs grown on graphene–RGD peptide nanoisland composite were 2.63- and 2.95-fold higher than on bare 3D GO-SiNPs after four weeks of differentiation. Other osteogenic differentiation indicators, such as ALP enzyme activity and calcium accumulation, were also highly enhanced, 1.48- and 1.58-fold higher than with bare 3D GO-SiNPs, proving the excellence of 3D graphene–RGD peptide nanoisland composite for steering ADSC osteogenesis. Moreover, ADSCs grown on the developed composites showed increased osteoblastic mineralization between days 14–21, 1.2-fold higher than on bare GO-SiNPs, exhibiting the potential of the developed platform to accelerate stem cell osteogenesis.

The 3D graphene–RGD peptide nanoisland composite shows great promise in stem cell research, and more advanced studies based on our findings will be required to improve the platform through the incorporation of different types of nanomaterials (e.g., polystyrene beads, glass beads) and peptides (cyclic RGD peptide, iRGD peptide). Other types of stem cells, such as pluripotent stem cells and neural stem cells, could also be applied to fully investigate the potential of 3D graphene–peptide nanoislands as a stable platform for long-term stem cell culture [66,67]. The desired cell types achieved from various stem cell lines using 3D graphene–peptide nanoisland composites as the culture platforms would then be usefully applied for clinical study, especially for the regeneration of damaged parts of human organs/tissues. Taken together, we can logically conclude that the developed platform, graphene–peptide complexes, could make remarkable contributions in the field of stem-cell-based regenerative medicine.

## 4. Materials and Methods

### 4.1. Materials

SiNPs were purchased from NanoComposix (San Diego, CA, USA). GO was obtained from Graphene Supermarket (Calverton, NY, USA). ADSCs and cell engineering for origin (CEFO) ADSC growth media supplemented with 10% fetal bovine serum and 1% penicillin/streptomycin were obtained from CEFO Bio (Seoul, Korea). Dexamethasone, L-ascorbic acid, β-glycerophosphate, and gold chloride trihydrate were purchased from Sigma Aldrich (St. Louis, MO, USA). RGD peptide was purchased from Peptron (Daejeon, Korea). ITO was purchased from UID (Sejong, Korea).

### 4.2. Fabrication of Three-Dimensional Hybrid Structures

First, ITO was prepared by sonication in 1% Triton X-100 in distilled water (DW) and pure ethanol for 20 min each. The ITO was spin-coated at 2000 rpm for 20 sec using 300 nm silica nanoparticles in 70% ethanol. After coating the substrates, 250 μg/mL GO solution was dropped onto the ITO and baked in a 70 °C oven for 1 h, after which the substrates were washed with DW. A chamber of 0.8 cm diameter was placed on the ITO substrates, using Polydimethylsilozane (PDMS) for cold deposition. A 0.5 mM gold solution was electrochemically treated at an initial voltage of −1.3 at 0, 40, 80, and 120 s. After electrical deposition, the chamber was washed with PBS, and coated with 0.05 mg/mL RGD peptide overnight at 4 °C.

### 4.3. Substrate Characterization

The silica-coated substrates were characterized by a variety of analytical techniques, including scanning electron microscopy (SEM, SIGMA, Carl Zeiss, Oberkochen, Germany), XPS (K-alpha+, Waltham, MA, USA; Thermo Fisher Scientific, Waltham, MA, USA.), and Raman spectroscopy.

### 4.4. Cell Proliferation and Spreading Analysis

ADSCs (6000 cells/cm^2^) were cultured on the 3D hybrid substrates in growth medium for 2 day. Before staining, cells were fixed in neutral buffered formalin (Sigma Aldrich) and permeabilized with 0.1% Triton X-100 for 5 min. After staining with Alexa Fluor 568 phalloidin (Life Technology, Carlsbad, CA, USA) and Hoechst 33342 (Thermo Scientific, Waltham, MA, U.S.A), cells were imaged by fluorescence microscopy. Cell proliferation and spreading were analyzed using ImageJ software.

### 4.5. Osteogenesis of ADSCs

The osteogenic differentiation medium contained 50 μM l-ascorbic acid, 10 mM β-glycerophosphate, and 100 nM dexamethasone. ADSCs were seeded in chambers, and the medium was changed every 2 days for 4 weeks. After the induction of osteogenesis, differentiation was confirmed by assaying calcium deposition with ARS. Cells were stained with ARS for 20 min, washed with DW, and imaged on an optical microscope. Subsequently, 10% cetylpyridinium chloride in 10 mM sodium phosphate was added, and the solution was incubated at 25 °C for 15 min. ARS extraction was measured at 562 nm on a microplate reader. In the case of alkaline phosphatase (ALP) analysis, ALP activity was measured based on the conversion of p-nitrophenyl phosphate (pNPP) into p-nitrophenol. First, the cell lysates achieved and were added in to 96 well plate with reaction reagent (pNPP). The plates were then incubated at 25 °C for 15 min while protecting the plates from light. Finally, the reaction was terminated with the addition of 3 M NaOH, followed by reading the absorbance at 405 nm using the plate reader.

### 4.6. Osteogenic Marker Expression Analysis

Total RNA was extracted from osteogenic cells using an RNeasy Mini Kit (Qiagen, Hilden, Germany) according to the manufacturer’s instructions, and cDNA was synthesized from 1 μg of total RNA with SuperScript II Reverse Transcriptase (Invitrogen, Carlsbad, CA, U.S.A). RT-qPCR was performed with SYBR Premix Ex Taq (Takara, Shiga, Japan) in a StepOnePlus RT-PCR system. 

## Supplementary Materials

Supplementary materials can be found at http://www.mdpi.com/1422-0067/19/3/669/s1.

## Abbreviations

ADSC	Adipose-derived mesenchymal stem cells
ALP	Alkaline phosphatase
ARS	Alizarin red S
DW	Distilled water
ECM	Extracellular matrix
ITO	Indium tin oxide
GNP	Gold nanoparticle
GO	Graphene oxide
PDMS	Polydimethylsilozane
RGD	Arginine-glycine-aspartic acid
RT-qPCR	Reverse transcription quantitative polymerase chain reaction
RUNX2	Runt-related transcription factor 2
SEM	Scanning electron microscopy
SiNPs	Silica nanoparticles
XPS	X-ray photoelectron spectroscopy
