# Supplementary material for: Three-Dimensional Graphene–RGD Peptide Nanoisland Composites That Enhance the Osteogenesis of Human Adipose-Derived Mesenchymal Stem Cells
Source: Int J Mol Sci. 2018 Feb 27;19(3):669. doi: 10.3390/ijms19030669 (PMC5877530; doi:10.3390/ijms19030669)
Supplement: Supplementary file 1 [file ijms-19-00669-s001.pdf]

Supplementary Information

TABLE OF CONTENTS

Figure S1. The XPS data of ITO/GO/SiNPs/Gold.
Figure S2. Raman spectroscopy spectra of each sample.
Figure S3. Fluorescence images of hADSCs grown on different substrates.
Figure S4. Optical microscopic images of hADSCs with growth medium.

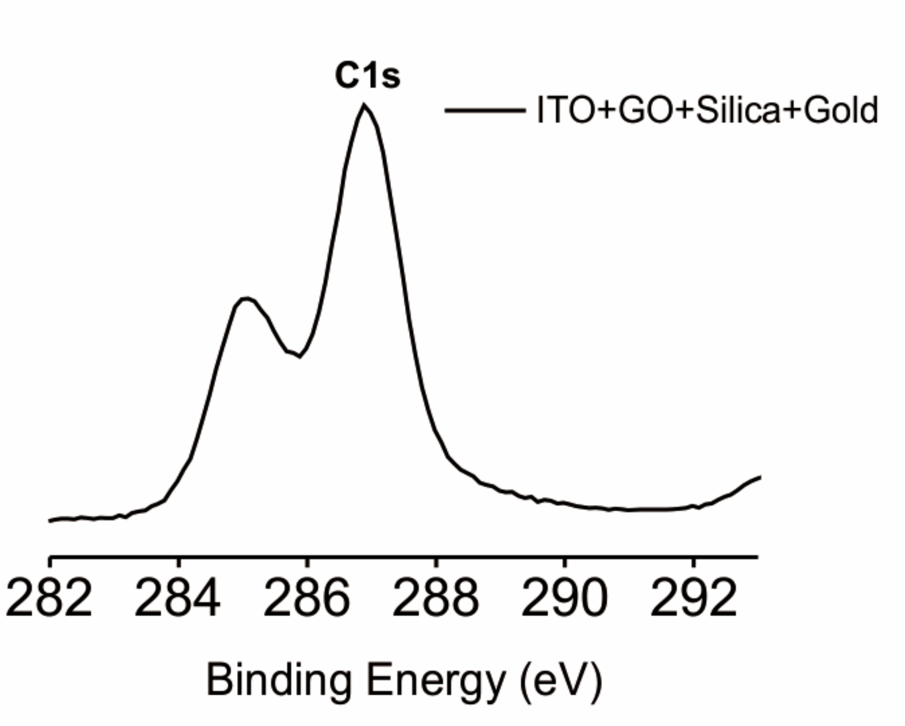

Figure S1. The XPS data of ITO/GO/SiNPs/GNPs.

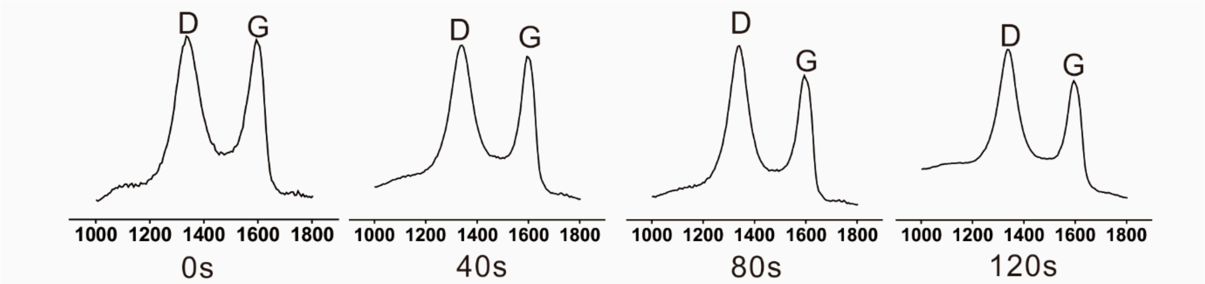

Figure S2. Raman spectroscopy spectra of each sample.

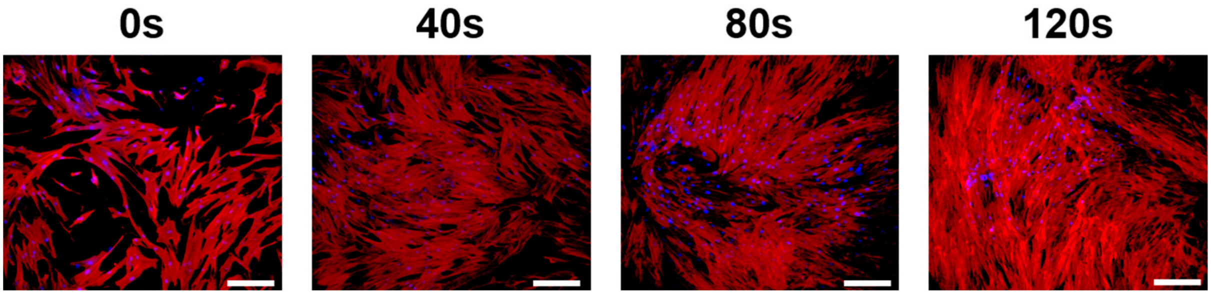

Figure S3. Fluorescence images of hADSCs grown on different substrates. F-Actin (red) and nucleus (blue) double-stained fluorescence images of hADSCs that were cultured on four different substrates. Images were used as source for cell growth and spreading analysis (Figure 4). (Scale bars = 200um).

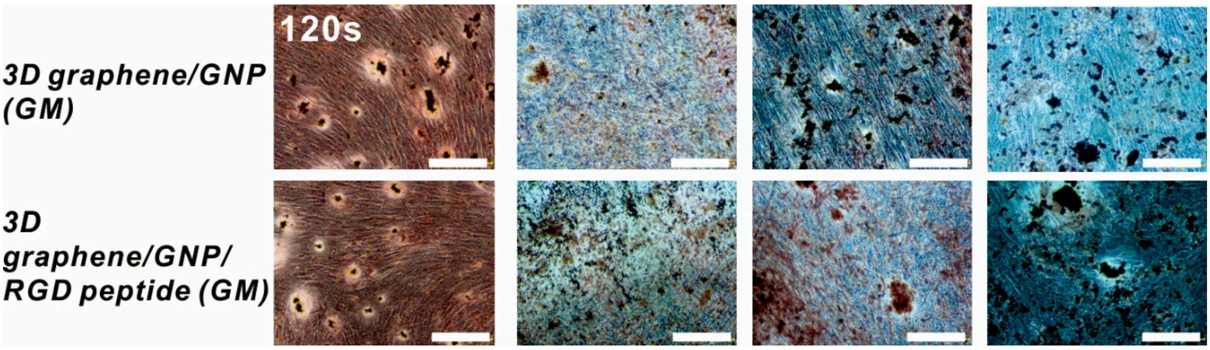

Figure S4. Optical microscopic images of hADSCs with growth medium. Confirmation of differentiation of hADSC growth on 3D nanoislands were revealed for 4 weeks. (Scale bar = 200μm).
